# Shared nociceptive dorsal root ganglion neurons participating in acupoint sensitization

**DOI:** 10.3389/fnmol.2022.974007

**Published:** 2022-08-29

**Authors:** Wanrong Li, Jia Liu, Aiwen Chen, Danqing Dai, Tiantian Zhao, Qiong Liu, Jianren Song, Lize Xiong, Xiao-Fei Gao

**Affiliations:** ^1^Translational Research Institute of Brain and Brain-Like Intelligence, Shanghai Fourth People’s Hospital, School of Medicine, Tongji University, Shanghai, China; ^2^Clinical Research Center for Anesthesiology and Perioperative Medicine, Tongji University, Shanghai, China; ^3^Department of Anesthesiology and Perioperative Medicine, Shanghai Fourth People’s Hospital, School of Medicine, Tongji University, Shanghai, China; ^4^Shanghai Key Laboratory of Anesthesiology and Brain Functional Modulation, Shanghai, China; ^5^Faculty of Anesthesiology, Changhai Hospital, Naval Medical University, Shanghai, China

**Keywords:** pain, dorsal root ganglion, acupoint sensitization, ST36 acupoint, nociceptive neuron, acupuncture

## Abstract

When the body is under pathological stress (injury or disease), the status of associated acupoints changes, including decreased pain threshold. Such changes in acupoint from a “silent” to an “active” state are considered “acupoint sensitization,” which has become an important indicator of acupoint selection. However, the mechanism of acupoint sensitization remains unclear. In this study, by retrograde tracing, morphological, chemogenetic, and behavioral methods, we found there are some dorsal root ganglion (DRG) neurons innervating the ST36 acupoint and ipsilateral hind paw (IHP) plantar simultaneously. Inhibition of these shared neurons induced analgesia in the complete Freund’s adjuvant (CFA) pain model and obstruction of nociceptive sensation in normal mice, and elevated the mechanical pain threshold (MPT) of ST36 acupoint in the CFA model. Excitation of shared neurons induced pain and declined the MPT of ST36 acupoint. Furthermore, most of the shared DRG neurons express TRPV1, a marker of nociceptive neurons. These results indicate that the shared nociceptive DRG neurons participate in ST36 acupoint sensitization in CFA-induced chronic pain. This raised a neural mechanism of acupoint sensitization at the level of primary sensory transmission.

## Introduction

Acupuncture and moxibustion are crucial components of traditional Chinese medicine (TCM). By applying physical stimulations, such as mechanical (acupuncture, pressing, etc.), thermal (moxibustion), or electrical (electroacupuncture, transcutaneous electrical nerve stimulation, etc.) stimuli, to certain specific points (acupoints) on the body surface, the relief of symptoms or treatment of diseases can be achieved. For example, a meta-analysis indicated that acupuncture had a beneficial effect on knee osteoarthritis in reducing pain and improved patient function activities (Tian et al., 2022). In the treatment of diarrhea-predominant irritable bowel syndrome, moxibustion had a more positive influence on the symptoms as compared to western medicines, TCM prescription, or acupuncture (Dai et al., 2022). In terms of improving immune function, studies have found that acupuncture significantly increased the numbers of white blood cells in patients with breast cancer who were undergoing ameliorating leukopenia during chemotherapy (Shih et al., 2021). At the same time, in the treatment of different diseases, some acupoints are often stimulated to receive better curative effects than others, so these acupoints are more favored. Acupoints such as ST36 (Zusanli) and EX-LE5 (Xiyan) were usually used in the treatments of knee osteoarthritis (Ma et al., 2018; Tong et al., 2021; Tian et al., 2022; Wei et al., 2022). To relieve diarrhea-predominant irritable bowel syndrome, ST36 and ST37 (Shangjuxu) were chosen with the highest frequency during the moxibustion treatments (Wang et al., 2018; Bao et al., 2019; Dai et al., 2022). Acupoints like ST36, SP6 (Sanyinjiao), BL23 (Shenyu), etc. were frequently used to improve immune function (Shih et al., 2018, 2021; Li et al., 2021; Oh and Kim, 2021; Ramires et al., 2021; Zhu et al., 2022). Therefore, to obtain a good therapeutic effect in acupuncture or moxibustion treatment, the selection of appropriate acupoints plays a key role.

It has been reported that when the body is under pathological stress (injury or disease), the status of associated acupoints changes, including sensation threshold, receptive field, biophysical properties, and morphology (Ngai et al., 2011; Wong, 2014; He et al., 2015; Zhu, 2015; Jung et al., 2017; Qi et al., 2017; Fan et al., 2018; Tan et al., 2019; Ma, 2021). In particular, acupoints undergo sensory mutations that increase sensitivity to various stimuli (similar to “allodynia”) (Zhu, 2019). Such changes in acupoint from a “silent” to an “active” state during pathological processes are considered “acupoint sensitization” (Tan et al., 2019). Selected sensitized acupoints for acupuncture treatment are in line with the account of “taking the tender point as acupoint” in *The Yellow Emperor’s Classics of Internal Medicine (Huang Di Nei Jing)*. For example, a recent trial study showed that acupressure at sensitized acupoints effectively reduced the frequency of stable angina pectoris episodes (Huang et al., 2021). Acupoint sensitization has become an important indicator of acupoint selection. However, the mechanism of acupoint sensitization remains unclear.

The neural system plays important role in the therapeutic effect of acupoints (Lee et al., 2020; Liu et al., 2020, 2021; Song et al., 2021; Li et al., 2022). Pain and heat sensitization is an important manifestation of acupoint sensitization, which also indicates the criticality of the sensory nervous system. The cell bodies of the sensory nerves innervating the acupoints gather in the dorsal root ganglia (DRG). In our previous study, we found that most DRG neurons innervating ST36 acupoint are middle-sized neurons with a single spike firing pattern, which suggests that proprioceptive neurons have a greater possibility than small-size nociceptive neurons to mediate the acupuncture signals (Li et al., 2022). However, small-diameter neurons are generally considered to mediate pain, itch, and heat sensations (Gao X. F. et al., 2015; Li et al., 2016; Zhang Z. J. et al., 2018; Dong et al., 2020). Therefore, it still needs to be elucidated which type of DRG neurons participate in acupoint sensitization.

In this study, we employed morphological, chemogenetic, and behavioral methods and explored the anatomical distribution and functional types of DRG neurons that mediate acupoint sensitization. We found that there were some DRG neurons innervating ST36 acupoint and ipsilateral hind paw (IHP) plantar simultaneously. Excitation of these shared neurons induced pain in normal mice, while inhibition of them elevated the mechanical pain threshold (MPT) of ST36 acupoint in the complete Freund’s adjuvant (CFA) model mice. TRPV1 channels, always expressed by nociceptive neurons, were also expressed by most of these shared neurons. These results indicate that the shared DRG neurons, most of which are nociceptive neurons, explain the neural mechanism of acupoint sensitization at the level of primary sensory transmission.

## Materials and methods

### Animals

Specific pathogen-free (SPF) grade C57BL/6J male mice (4–8 weeks old) were purchased from the Shanghai Tongji Biotechnology Co., Ltd. (Shanghai, China). All mice were kept in a room with controlled temperature (25 ± 1°C) and humidity (55 ± 5%), and 12 h (8:00 a.m. to 8:00 p.m.) light–dark cycle with free access to water and rodent chow. All mice were group-housed (4–6 per cage) to avoid social isolation stress. All experimental protocols were approved by the Ethics Committee of the Laboratory Animal Center of Tongji University (Approval Number: TJBH05621101) and performed following the Guide for the Care and Use of Laboratory Animals of Tongji University (Shanghai, China) and the International Guide for the Care and Use of Laboratory Animals.

### Retrograde tracer injections

Retrograde tracer in this study included Fluoro-Gold (FG, Fluorochrome Inc., Englewood, CO, United States) and the lipophilic tracer, 1′-dioctadecyl-3,3,3′,3′-tetramethylindocarbocyanine perchlorate (DiI, D282; Invitrogen, Thermo Fisher Scientific, Waltham, MA, United States) (Chang et al., 2016). FG was dissolved in 0.9% saline to yield a 4% concentration (Szawka et al., 2013; Pattwell et al., 2016). DiI was dissolved in dimethyl sulfoxide (DMSO, D2650; Sigma-Aldrich, St. Louis, MO, United States) as a stock solution, and freshly prepared in 0.9% saline.

During DiI stereotaxic injections, the mice were fixed in mouse pockets and held in the appropriate position of the stereotaxic apparatus. The left leg that needed to be injected was secured to the outer wall of the mouse pocket. The injection site was ST36 acupoint, so fur around ST36 acupoint needs to be shaved to expose the skin. ST36 acupoint is 2–3 mm below the small head of the fibula and lateral to the anterior tubercle of the tibia (Zhang K. et al., 2018; Lin et al., 2019). According to the location of the ST36 acupoint, the tip of the injection needle was first positioned on the skin surface of the small head of the fibula as the starting points (lateral 0 mm, longitudinal 0 mm, and depth 0 mm). Then, 5 μL of DiI (2 mg/mL) was injected unilaterally into ST36 acupoint (lateral 2.5 mm, longitudinal 0 mm, and depth 3 mm) at a flow rate of 1 μL/min by a monitored microsyringe. The needle stayed at ST36 acupoint for an extra 1 min before being withdrawn slowly. Ten days after DiI injection, 1.5 μL FG was injected into the plantar surface of the left hind paw of mice. Two weeks after DiI injection and 4 days after FG injection, lumbar 4 to lumbar 6 (L4–L6) DRGs were dissected for immunofluorescence. A total of 15 mice were used in this experiment.

### Chemogenetic method

Viruses utilized in this study included AAVRetro-hSyn-DIO-hM4D(Gi)-mCherry (titer 1.060 × 10^12^ virus particles/mL), AAVRetro-hSyn-DIO-hM3D(Gq)-mCherry (titer 1.135 × 10^12^ virus particles/mL), AAVRetro-hSyn-DIO-mCherry (titer 1.163 × 10^12^ virus particles/mL), and AAVRetro-hSyn-Cre-EGFP (titer 1.285 × 10^12^ virus particles/mL), all of which were obtained from the Shanghai Genechem Technology Co., Ltd. Viruses were injected 3–4 weeks before behavioral tests.

ST36 acupoint stereotaxic injection was performed as previously described. Eight microliters of AAVRetro-hSyn-DIO-hM4D(Gi)-mCherry, AAVRetro-hSyn-DIO-hM3D(Gq)-mCherry or AAVRetro-hSyn-DIO-mCherry was injected into left ST36 acupoint (lateral 2.5 mm, longitudinal 0 mm, sand depth 3 mm) at a flow rate of 1.5 μL/min. And 8 μL of AAVRetro-hSyn-Cre-EGFP was injected into the plantar surface of the left hind paw of mice who were fixed in mouse pockets during the injection. During all injections, the needle was allowed to remain at the target site for an additional 10 min and then removed slowly to prevent efflux of the virus. All virus injections were completed on the same day. A total of 85 mice received virus injections.

For hM4Di silencing or hM3Dq activation, mice were injected clozapine-N-oxide (CNO, C0832, Sigma-Aldrich) intraperitoneal (i.p.) at 5 mg/kg, 30 min before the behavioral test (Stoodley et al., 2017; Liu et al., 2020). CNO was dissolved in DMSO at 10 mg/mL as a stock solution, kept at – 20°C until used, and freshly diluted with 0.9% saline to the corresponding working concentrations in a final volume of 300 μL before intraperitoneal administration. The final concentration of DMSO in working solutions did not exceed 0.1% (vol/vol). Saline was injected as vehicle control.

### Complete Freund’s adjuvant model

Fifty mice received CFA injections. CFA was purchased from Sigma-Aldrich (F5881), which contains 1 mg/mL of heat-killed and dried mycobacterium tuberculosis in 85% paraffin oil and 15% mannide monooleate. Unilateral inflammation pain was induced by injecting 20 μL CFA into the plantar surface of the left hind paw of mice who were fixed in mouse pockets during the injection (Wu et al., 2019). Once finished injection, the injection site was immediately pressed gently with cotton to prevent bleeding and drug leakage.

## Behavioral tests

Behavioral tests were performed on 97 mice, of which 12 mice were measured before and 7 days after CFA injection to confirm the CFA model was built successfully. Three to four weeks after virus injection, mice were measured before or 4 days after CFA injection (Goldman et al., 2010). During the subsequent studies, mice were first injected with saline (vehicle-only i.p. injection), followed by behavioral testing 30 min later (CNO OFF state). And then, the mice were injected with CNO (i.p. injection), followed by behavioral testing another 30 min later (CNO ON state). The results of each test were paired for comparison as a vehicle control group (saline) and a treatment group (CNO). Throughout the study, all criteria for behavioral tests that followed were determined *a priori*. All behavioral testing was performed between 8 a.m. and 12 a.m. Before experiments, mice were randomly assigned to groups. Researchers involved in behavioral testing and data analysis were blinded to the group assignment. Mice were habituated to the test environment for 30 min daily for successive 3 days before the behavioral testing. And before the initiation of each test session, mice were allowed to acclimate to the testing apparatus for 30 min. Three trials were performed per mouse with an interval between measurements of at least 5 min, and the average of the 3 trials was taken as the result.

**FIGURE 1:**
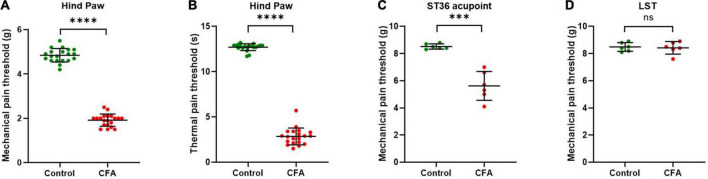
ST36 acupoints were sensitized in CFA model mice. **(A)** The mechanical pain threshold of the hind paw decreased in the CFA model. *n* = 20, ^****^P < 0.0001, paired *t*-test. **(B)** The thermal pain threshold of the hind paw decreased in the CFA model. *n* = 20, ^****^P < 0.0001, paired *t*-test. **(C)** The mechanical pain threshold of ST36 acupoints decreased in the CFA model. *n* = 6, ^***^P = 0.0009, paired *t*-test. **(D)** The mechanical pain threshold of LST did not change significantly in the CFA model. *n* = 6, *P* = 0.7412, paired *t*-test. LST, the position 5 mm lateral side of the ST36.

Behavioral data in Figures 1A,B (*n* = 20) were collected from the same batch of mice in Figure 3B (*n* = 9) and Figure 5B (*n* = 11). And behavioral data in Figures 1C,D (*n* = 6) were collected from the same batch of mice in Figure 4A (*n* = 6).

**FIGURE 2:**
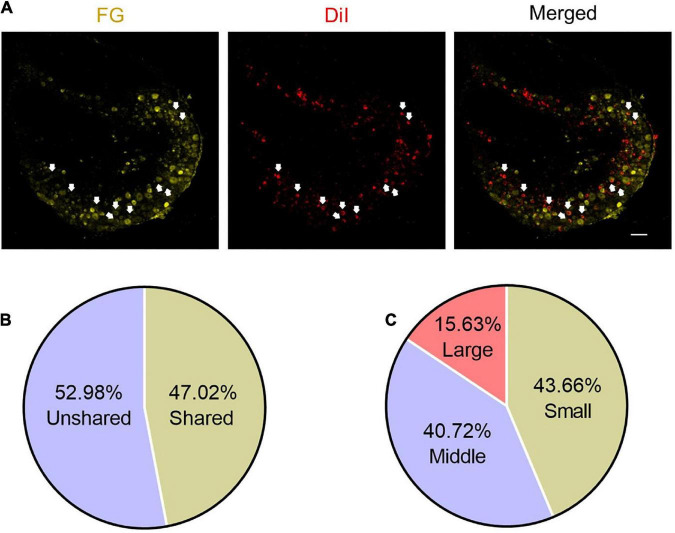
Some DRG neurons innervate both ST36 acupoint and ipsilateral hind paw plantar. **(A)** The representative pictures of shared DRG neurons. FG, Fluoro-Gold. The white arrows indicate double-labeled DRG neurons. Bar = 100 μm. **(B)** The percentage of shared neurons in all DRG neurons. **(C)** The percentages of neurons with different diameters in shared neurons.

**FIGURE 3:**
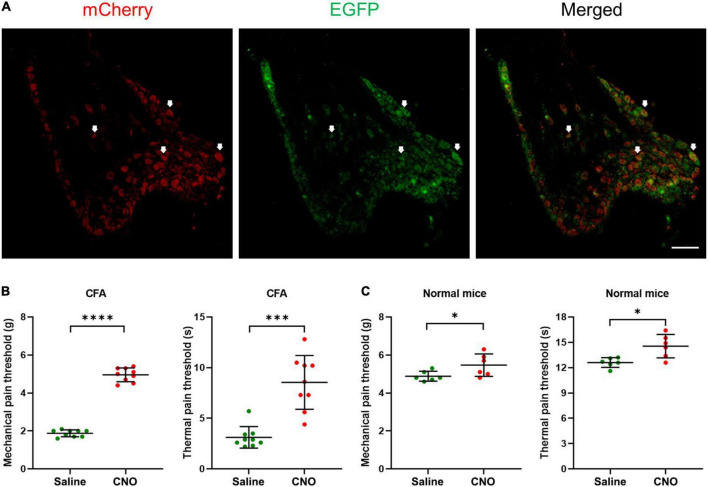
Inhibition of shared neurons induced analgesia in CFA mice. **(A)** The representative pictures of DRG neurons expressing hM4D(Gi) receptors. The white arrows indicate double-labeled DRG neurons. As the EGFP signals were from original EGFP proteins expressed by the AAVRetro-hSyn-Cre-EGFP virus, while the mCherry proteins were expressed in a cre-dependent manner and their signals were amplified by the mCherry antibodies, all mCherry signal positive neurons were shared neurons. Bar = 150 μm. **(B)** The mechanical pain thresholds and thermal pain thresholds of the ipsilateral hind paw plantar were elevated after CNO treatment in the CFA model. *n* = 9, paired *t*-test. For mechanical pain thresholds, ^****^P < 0.0001. For thermal pain thresholds, ^***^P = 0.0004. **(C)** The mechanical pain thresholds and thermal pain thresholds of the ipsilateral hind paw plantar were increased a little after CNO treatment in normal mice. *n* = 6, paired *t*-test. For mechanical pain thresholds, *P = 0.0472. For thermal pain thresholds, *P = 0.0122.

**FIGURE 4:**
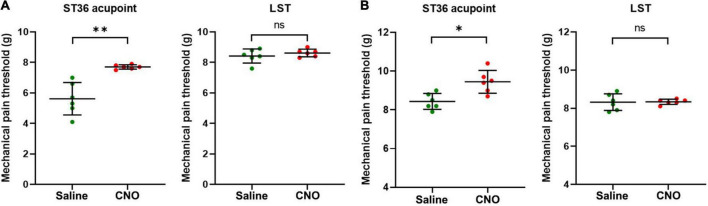
Inhibition of shared neurons elevated the mechanical pain threshold of ST36 acupoint. **(A)** The CNO treatment increased the mechanical pain thresholds of the ipsilateral ST36 acupoint but not the LST in the CFA model. *n* = 6, paired *t*-test. For ST36 acupoint, ^**^P = 0.0032. For LST, *P* = 0.3632. LST, the position 5 mm lateral side of the ST36. **(B)** The CNO treatment increased the mechanical pain thresholds of the ipsilateral ST36 acupoint but not the LST in the normal mice. *n* = 6, paired *t*-test. For ST36 acupoint, *P = 0.0284. For LST, *P* = 0.9343.

**FIGURE 5:**
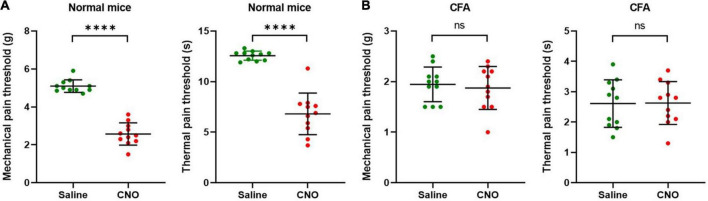
Activation of shared neurons induced pain. **(A)** The mechanical pain thresholds and thermal pain thresholds of the ipsilateral hind paw plantar were decreased after CNO treatment in normal mice. *n* = 11, paired *t*-test. For mechanical pain thresholds, ****P < 0.0001. For thermal pain thresholds, ****P < 0.0001. **(B)** The mechanical pain thresholds and thermal pain thresholds of the ipsilateral hind paw plantar did not change significantly after CNO treatment in the CFA model. *n* = 11, paired *t*-test. For mechanical pain thresholds, *P* = 0.7334. For thermal pain thresholds, *P* = 0.9560.

### Mechanical pain sensitivity

Mechanical pain threshold (MPT) was assessed with the electronic Von Frey test (BIO-EVF4, Bioseb, Vitrolles, France) at three locations separately, ST36 acupoint, 5 mm lateral side of ST36 acupoint (LST), and plantar of the IHP. To measure the MPT of IHP, each mouse was placed in a suspended plastic cage (66 × 22 × 13 cm) with a wire grid floor (0.6 × 0.6 cm^2^ spacing). And to measure the MPT of ST36 acupoint or LST, the mice were fixed in mouse pockets. The electronic von Frey apparatus, consisting of an elastic spring-type tip fitted in a hand-held force transducer, was applied perpendicularly to the skin surface of the three locations and the force applied was gradually increased until hind paw withdrawal. Finally, MPT (expressed in grams) was automatically recorded by the electronic Von Frey device and regarded as a pain parameter (Ferrier et al., 2013).

### Thermal pain sensitivity

Thermal pain threshold (TPT) was evaluated with a thermal pain test apparatus (Hargreaves Apparatus, No. 37370, Ugo Basile, Comerio, Italy). Before testing, mice were individually kept in a transparent plastic test chamber (62 × 20 × 14 cm) fixed in the proper position on a suspended, specially constructed, and non-insulated transparent glass panel (86 × 35 cm). The intensity of the infrared radiant heat source (IR value) was adjusted and kept constant. And to prevent possible tissue damage, the basal TPT was adjusted to 10–12 s and a cutoff of 20 s (Mao-Ying et al., 2012; Feng et al., 2019). In this study, a movable stimulus of infrared radiant heat (IR = 23) was placed under the glass floor directly beneath the middle plantar surface of the left hind paw. When the withdrawal response occurred, the IR source switched off, and the reaction time counter stopped. The TPT was automatically detected and recorded (in seconds) with an accuracy of 0.1 s.

### Immunofluorescence staining

DRGs (L4-L6) were harvested from 15 mice perfused with ice-cold PBS and 4% paraformaldehyde (PFA). DRGs were fixed at 4°C in 4% PFA for at least 2 h, incubated in 30% sucrose overnight at 4°C, and embedded in optimal cutting temperature compound (OCT, 4583, Tissue-Tek, SAKURA, Torrance, CA, United States). Coronal sections (30 μm thick) were cut with a cryomicrotome (CM 1950, Leica Microsystems, Nussloch, Germany), and adhered to slides. For staining, sections were washed in PBS three times (3 min for each wash), blocked in PBS containing 5% normal goat serum for 2 h at room temperature, and incubated with primary antibodies overnight at 4°C. Primary antibodies utilized in this study included rabbit polyclonal anti-transient receptor potential vanilloid 1 (TRPV1) antibody (1:400 dilution, ab6166, Abcam, Cambridge, United Kingdom), and rabbit monoclonal anti-Parvalbumin (PV) Antibody (1:200 dilution, MA5-35259, Thermo Fisher Scientific). After three washes with PBS for 3 min each time, the sections were incubated with goat polyclonal secondary antibody to rabbit IgG-H&L (Alexa Fluor^®^ 488, 1:500 dilution, ab150077, Abcam) for 2 h at room temperature. Finally, sections were washed in PBS three times (3 min for each wash) and sealed with an appropriate amount of antifading mounting reagent (S2100, Solarbio, Shanghai, China). The images were captured by confocal laser-scanning microscopy using an Olympus FluoView FV3000 self-contained confocal laser scanning microscope system (Olympus, Tokyo, Japan). The data of immunofluorescent staining presented in the Figures 2A, 3A, 7A,D are representative images of at least five mice (Rezende et al., 2017; Lavian and Korngreen, 2019).

**FIGURE 6:**
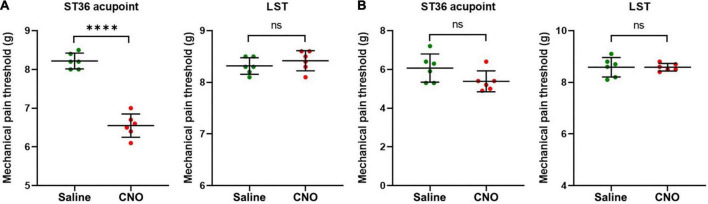
Activation of shared neurons declined the mechanical pain threshold of ST36 acupoint. **(A)** The CNO treatment decreased the mechanical pain thresholds of the ipsilateral ST36 acupoint but not the LST in the normal mice. *n* = 6, paired *t*-test. For ST36 acupoint, ^****^P < 0.0001. For LST, *P* = 0.4466. LST, the position 5 mm lateral side of the ST36. **(B)** The CNO treatment did not significantly change the mechanical pain thresholds of the ipsilateral ST36 acupoint and the LST in the CFA model. *n* = 6, paired *t*-test. For ST36 acupoint, *P* = 0.1849. For LST, *P* > 0.9999.

**FIGURE 7:**
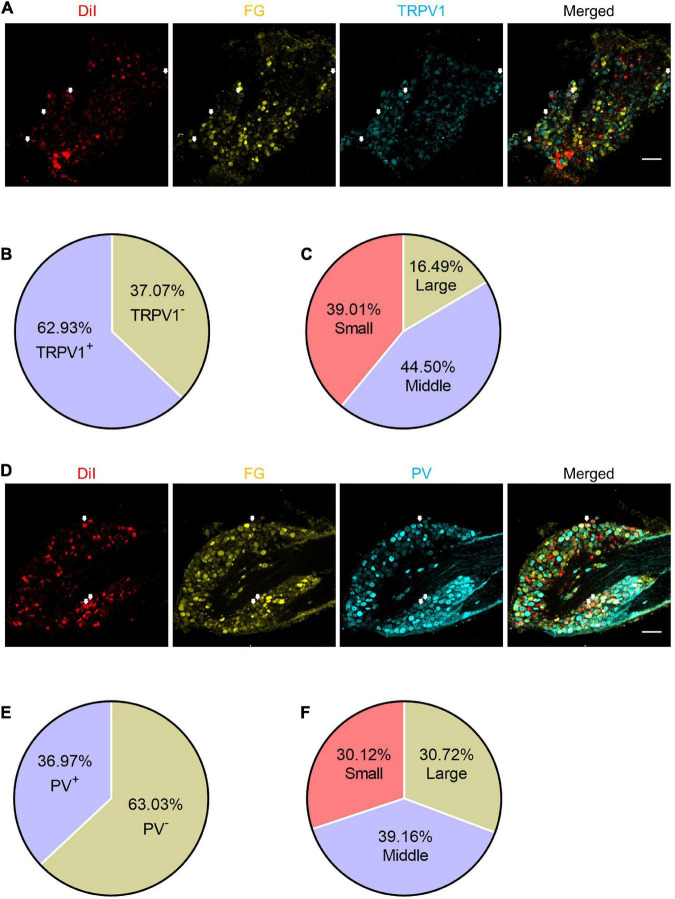
The expression patterns of TRPV1 and PV in the shared DRG neurons. **(A)** The representative pictures of expression of TRPV1 in shared DRG neurons. FG, Fluoro-Gold. The white arrows indicate triple-labeled DRG neurons. Bar = 150 μm. **(B)** The percentage of TRPV1 positive neurons in shared neurons. **(C)** The percentages of TRPV1 positive shared neurons with different diameters. **(D)** The representative pictures of expression of PV in shared DRG neurons. The white arrows indicate triple-labeled DRG neurons. Bar = 150 μm. **(E)** The percentage of PV positive neurons in shared neurons. **(F)** The percentages of PV positive shared neurons with different diameters.

### Statistics

The statistical analysis was performed using the Graphpad Prism 8 software (GraphPad, San Diego, CA, United States). All data are presented as mean ± standard deviation (SD) and checked for normality before analysis. For comparison of parameters between two groups (Figures 1, 3B,C, 4–6), the variance similarity was determined by the *F*-test. And significance was determined by paired *t*-test. For all tests, differences of *P* < 0.05 were considered to be statistically significant. Statistical analysis of individual experiments is also described in figure legends.

## Results

### The mechanical pain threshold of ST36 acupoint was decreased in complete Freund’s adjuvant-treated mice

The acupoint being more sensitive to external mechanical stimuli is one of the characteristics of acupoint sensitization. After the CFA model was confirmed to be built successfully (Supplementary Figure 1), we checked the pain threshold of hind paw plantar in the CFA-treated mice. After 4 days of CFA injection into the hind paw plantar, the MPTs of the hind paws were decreased from 4.8 ± 0.3 g to 1.9 ± 0.3 g (Figure 1A), and the TPTs were reduced from 12.7 ± 0.4 s to 2.8 ± 0.9 s (Figure 1B). These indicate that we achieved a stable CFA pain model. Then we evaluated the MPTs of ipsilateral ST36 acupoints in these CFA model mice by the von Frey method. CFA treatment significantly reduced the MPTs from 8.4 ± 0.4 g to 5.6 ± 1.1 g (Figure 1C), while the thresholds of the position 5 mm lateral side of the ST36 (LST) did not change much, from 8.3 ± 0.4 g to 8.4 ± 0.5 g (Figure 1D). These data suggested that ST36 acupoint was sensitized in the CFA-induced inflammatory pain state.

### Dorsal root ganglion neurons shared by ST36 acupoint and ipsilateral hind paw plantar

Since the sensory afferents of the calf and foot are the component of the sciatic nerve, and our previous study also showed that the somata of the nerve terminals innervating the ST36 acupoint were distributed in the L4–L6 DRG (Li et al., 2022), we hypothesized that there may be DRG neurons innervating both the plantar and ipsilateral ST36 acupoint in naïve mice. We retro-labeled DRG neurons innervating ST36 acupoint and ipsilateral plantar by injecting DiI or FG dyes at these two sites, respectively (Figure 2A). Since we are more concerned about how many neurons related to ST36 acupoint belong to shared neurons, we used the number of DiI-positive neurons as the denominator and the number of double-labeled neurons as the numerator when calculating the percentage, that is (DiI^+^ FG^+^)/DiI^+^ × 100%. After statistical analysis, we found that about 47.02% (1,056/2,246) of DRG neurons were double-labeled by DiI and FG, which indicated that these neurons were shared by ST36 acupoint and IHP plantar (Figure 2B). Of these shared neurons, 43.66% (461/1,056) were small-diameter neurons, 40.72% (430/1,056) were middle-size neurons, and only 15.63% (165/1,056) were large-diameter neurons (Figure 2C).

### Inhibition of shared neurons induced analgesia

To understand the potential function of shared DRG neurons, we injected the AAVRetro-hSyn-Cre-EGFP virus into hind paw plantar and AAVRetro-hSyn-DIO-hM4D(Gi)-mCherry virus into ipsilateral ST36 acupoint at the same time to induce the expression of Gi-coupled human M4 muscarinic DREADD receptors in the shared DRG neurons. By immunofluorescence method, mCherry signals were detected in many DRG neurons (Figure 3A), which indicated that two viruses infected DRG terminals at two separate positions were retrogradely transported to the same soma, and expressed mCherry in a cre-dependent manner. Then CNO was injected intraperitoneally to specifically inhibit shared DRG neurons. We compared the MPTs and the TPTs after CNO treatment with those before CNO treatment and found that, in the CFA model, both thresholds were elevated significantly (MPTs from 1.9 ± 0.2 g to 5.0 ± 0.4 g, TPTs from 3.1 ± 1.1 s to 8.5 ± 2.7 s) (Figure 3B). Even in normal mice, the thresholds were also increased a little (MPTs from 4.9 ± 0.3 g to 5.5 ± 0.6 g, TPTs from 12.6 ± 0.6 s to 14.5 ± 1.4 s) (Figure 3C). These data suggest that selectively inhibition of shared DRG neurons induced analgesic effect in the CFA pain model and obstruction of nociceptive sensation in normal mice. We also performed the same studies as shown in Figure 3, except that the AAVRetro-hSyn-DIO-hM4D(Gi)-mCherry virus was replaced by the AAVRetro-hSyn-DIO-mCherry virus, which means that only the mCherry proteins, but not M4 muscarinic DREADD receptors, were expressed in shared DRG neurons. The data showed that CNO did not change the pain thresholds of plantar both in the CFA model and in normal mice (Supplementary Figures 2A,B), which indicates that the change of pain thresholds was dependent on the expression of M4 muscarinic DREADD receptors in the shared DRG neurons, in other words, the inhibition of shared DRG neurons.

### Inhibition of shared neurons elevated the mechanical pain threshold of ST36 acupoint

As the shared DRG neurons innervate both hind paw plantar and ST36 acupoint, they may be involved in the sensation of both locations. We hypothesize that inhibition of these neurons might also block the pain sensation of the ipsilateral ST36 acupoint as they did of the hind paw. In the CFA model, the MPTs of ST36 elevated from 5.0 ± 1.1 g to 7.7 ± 0.1 g by CNO treatment, while those of the LST position did not change much, from 8.4 ± 0.5 g to 8.6 ± 0.2 g (Figure 4A). In normal mice, the thresholds of LST also did not change by CNO, from 8.3 ± 0.4 g to 8.3 ± 0.1 g, but those of the ST36 still increased a little, from 8.4 ± 0.4 g to 9.4 ± 0.6 g (Figure 4B). The changing trend of MPTs at the ST36 acupoint was quite like those at the ipsilateral plantar.

### Excitation of shared neurons induced pain

The results of inhibition of shared DRG neurons indicate that these neurons are necessary conditions for, or at least involved in, transmitting nociceptive pain information from paw plantar or ST36 acupoint to the spinal cord. Then, we performed similar chemogenetic experiments to activate the shared DRG neurons specifically and evaluated the pain thresholds of the hind paw. The AAVRetro-hSyn-Cre-EGFP virus and AAVRetro-hSyn-DIO-hM3D(Gq)-mCherry virus were injected into hind paw plantar and ipsilateral ST36 acupoint separately at the same time. The CNO was injected intraperitoneally to specifically excite shared DRG neurons. The MPTs and the TPTs after CNO treatment were compared with those before CNO treatment. In normal mice, both thresholds declined significantly (MPTs from 5.1 ± 0.3 g to 2.6 ± 0.6 g, TPTs from 12.6 ± 0.5 s to 6.8 ± 2.1 s) (Figure 5A). These data suggest that selective activation of shared DRG neurons was sufficient to induce pain. But in the CFA model, the thresholds were not changed much (MPTs from 1.9 ± 0.3 g to 1.9 ± 0.4 g, TPTs from 2.6 ± 0.8 s to 2.6 ± 0.7 s) (Figure 5B).

### Excitation of shared neurons declined the mechanical pain threshold of ST36 acupoint

Furthermore, we examined the change in the MPT of ST36 acupoint when shared DRG neurons were activated by CNO. In normal mice, the MPTs of ST36 declined from 8.2 ± 0.2 g to 6.6 ± 0.3 g by CNO treatment, while those of the LST position did not change much, from 8.3 ± 0.2 g to 8.4 ± 0.2 g (Figure 6A). In the CFA model, the thresholds of LST also did not change by CNO, from 8.6 ± 0.4 g to 8.6 ± 0.1 g, while those of the ST36 still decreased a little, from 6.1 ± 0.7 g to 5.4 ± 0.5 g (Figure 6B). The changing trend of the MPTs at ST36 acupoint was also quite like those at the ipsilateral plantar.

We also performed the same studies as in Figure 6, except that the AAVRetro-hSyn-DIO-hM3D(Gq)-mCherry virus was replaced by the AAVRetro-hSyn-DIO-mCherry virus, which means that only the mCherry proteins, but not M3 muscarinic DREADD receptors, were expressed in shared DRG neurons. The data showed that CNO did not change the pain thresholds of ST36 acupoint or LST both in normal mice and in the CFA model (Supplementary Figures 3A,B), which indicates that the change of pain thresholds of ST36 acupoint in normal mice was dependent on the expression of M3 muscarinic DREADD receptors in the shared DRG neurons, in other words, the activation of shared DRG neurons.

### Most of the shared dorsal root ganglion neurons were nociceptive neurons

Based on the cell-size ratio of shared DRG neurons, most neurons were small to middle size. And the results of chemogenetic experiments strongly suggest that they are necessary and sufficient for the transmission of pain signaling. Thus, we stained the DRG slices with TRPV1 or PV antibodies to verify the expression properties of these neurons. About 62.93% (382/607) of shared DRG neurons were TRPV1^+^ neurons (Figures 7A,B), of which about 83.51% neurons were small (149/382) or middle (170/382) size neurons (Figure 7C). About 36.97% (166/449) of shared DRG neurons were PV^+^ neurons (Figures 7D,E). The 69.88% of PV^+^ neurons were large (51/166) or middle (65/166) size neurons (Figure 7F). These results went along that most of the shared DRG neurons were nociceptive neurons.

## Discussion

The mechanical and TPTs of the sensitized acupoints decreased, which is very similar to that of the specific areas of the body surface in visceral pain, such as the preferred pain on the body surface in angina pectoris (Foreman et al., 2015; Gebhart and Bielefeldt, 2016). The myofascial trigger point is quite a sensitized acupoint in knee-joint pain (Dorsher, 2008; Qin et al., 2020). In our study, we also found sensitization of ST36 acupoints in the CFA pain model. These results suggest that sensitization of the corresponding acupoints is a common phenomenon in both visceral pain and superficial pain. A similar neural mechanism may be shared by these two pain states.

Since the change of the pain thresholds of the plantar and ST36 acupoint showed the same trend (Figure 1), we hypothesized that there might be the same group of neurons to sense pain in these two places. Compared to the hypothesis that CFA leads to the emergence of new anatomical structures, the explanation that the existence of shared neurons under physiological conditions is simpler. Based on the principle of simplification, we first examined the existence of shared neurons in naïve mice. Fortunately, we did find neurons that were co-labeled with both dyes (Figure 2A). Furthermore, excitation of shared neurons declined the MPT of ST36 acupoint (Figure 6A), which mimics that the MPT of ST36 acupoint was decreased in CFA-treated mice (Figure 1C). Of course, we cannot exclude the possibility that CFA may also influence the terminal distribution of DRG neurons, but we believe our findings are also an important explanation for acupoint sensitization.

By chemogenetic methods, we specifically inhibited shared DRG neurons and found that the MPTs and the TPTs increased significantly in the CFA pain model (Figure 3B), which suggested the shared DRG neurons are necessary conditions for pain sensation of plantar. On the other hand, both thresholds declined significantly in normal mice when the shared DRG neurons were activated selectively (Figure 5A), which suggested that these neurons are also sufficient conditions for the pain sensation of plantar. The same trends of pain thresholds of ST36 acupoint were also detected when the shared DRG neurons were selectively modulated by CNO (Figures 4A,B, 5A,B). Thus, the shared DRG neurons are both necessary and sufficient conditions for pain transmitting of plantar and ST36 acupoint. In normal mice, inhibition of shared DRG neurons did not elevate the MPTs and TPTs dramatically (Figure 3C). This may be because in the saline control group, the pain induced by the von Frey stimulation itself was very weak and the shared DRG neurons were still relatively quiet. Therefore, selective inhibition of shared neurons by CNO could only raise the pain thresholds relatively limited. On the contrary, in the CFA model, the shared DRG neurons had been activated much in the saline control group. Selective activation of shared neurons by CNO could not further decrease the pain thresholds anymore (Figure 5B).

Recently, some DRG neurons have been reported to innervate both the skin and the distal colon simultaneously (Fang et al., 2020). *In vivo* extracellular electrophysiological recordings of those DRG neurons revealed that they robustly respond to both expansions of the distal colon and the mechanical, nociceptive, warm, or cold stimulation of the cutaneous receptive field (Fang et al., 2020). These results indicate that DRG neurons shared by both the skin and the distal colon participate in the transmission or modulation of multiple sensation signals. In this study, we also found some DRG neurons shared by ST36 acupoint and IHP plantar. The similar neuroanatomy again strongly suggests that the two groups of shared neurons may underlie the common mechanistic basis of the referred skin pain (the acupoint sensitization) in the visceral pain and superficial pain. Therefore, by morphological, chemogenetic, and behavioral methods, we further confirmed that the shared neurons we found were nociceptive and highly correlated with the decrease in pain threshold of ST36 acupoint. Our behavioral results are consistent with their extracellular electrophysiological findings, both indicating that shared neurons are involved in pain transmission.

**FIGURE 8:**
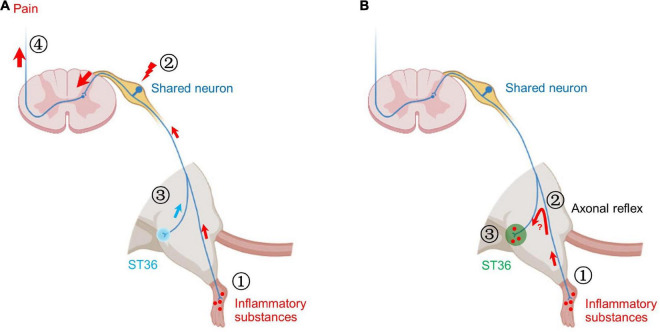
Two possible mechanisms of shared neurons participating in acupoint sensitization. **(A)** ➀ The inflammatory substances induced by CFA in the hind paw plantar stimulate the terminals of shared neurons. ➁ The pain signals are continuously transmitted to the somata of shared DRG neurons, resulting in increased neuronal excitability. ➂ Mechanical stimulation of ST36 more readily activates shared neurons. ➃ More pain signals are transmitted to the brain. Behaviorally, the pain threshold of ST36 acupoint decreased. **(B)** ➀ The inflammatory substances induced by CFA in the hind paw plantar stimulate the terminals of shared neurons. ➁ By the axonal reflex mechanism, the nerve impulses are transmitted to ST36 acupoint *via* axon bifurcation. ➂ The persistent nerve impulses promote the release of pro-inflammatory molecules from the nerve terminal and recruit mast cells to aggregate, degranulate, etc. Locally enhanced neuroimmune responses lead to lower pain thresholds and enlarged receptive field of ST36 acupoint.

Our previous study also found that the excitability of C-type DRG neurons innervating the sensitized ST35 (Dubi) acupoint was increased in the knee osteoarthritis rat model (Zhang et al., 2019). Based on the above findings, we believe that the increased excitability of shared nociceptive neurons that innervate both lesions and acupoints is one of the neural mechanisms leading to the acupoints sensitization, at least at the level of primary afferent neurons. In this study, the inflammatory substances induced by CFA in the hind paw plantar continue to stimulate the terminals of shared neurons, resulting in increased neuronal excitability, which makes it easier to transmit signals of mechanical stimuli from ST36 acupoint upward (Figure 8A; Prato et al., 2017; Zhang et al., 2019).

Many works of literature have reported that the efficacy of acupuncture is related to the C-type fibers innervating acupoints or DRG neurons expressing TRPV1 (Wang et al., 1996; Tjen et al., 2005; Qin et al., 2014; Gao X. et al., 2015; Chen et al., 2018; Guo et al., 2018; Liu et al., 2018; Li et al., 2019; Du et al., 2021; Fang et al., 2021; Song et al., 2021). However, the TRPV1-expressing shared DRG neurons in this study are unlikely to be involved in the analgesia produced by acupuncture at the ST36 acupoint. Because these neurons facilitate pain sensation of the hind paw. It is logically contradictory if the same neuron mediates both pain and analgesia at the same time. Or there may be some complex signal modulation mechanisms in the DRG or spinal cord to decide what kind of signal to pass up, but these still need further experiments to prove. In addition, there is also a lot of literature claiming that type A fibers are also involved in the analgesia of acupuncture (Katz et al., 1989; Leung et al., 2005; Kagitani et al., 2010; Lee et al., 2020; Qu et al., 2020). And our previous study reported that proprioceptive neurons are more likely to receive stimulation at acupoints by acupuncture (Li et al., 2022). Therefore, the neurons that receive acupuncture stimulation at acupoints and produce therapeutic effects may not be the same group of neurons that participate in acupoint sensitization.

Our behavioral studies revealed the role of shared neurons in the decreased pain threshold of sensitized acupoints, but cannot explain other immunological phenomena at sensitized acupoints yet, such as degranulation of local mast cells. The axonal reflex mechanism may be an appropriate explanation (Carlsson, 2002). The persistent pain signal from the hind paw may be transmitted retrogradely to the ST36 acupoint through the branch of afferent fibers, thereby promoting the release of pro-inflammatory molecules from the nerve terminal at the acupoint (Choi and Di Nardo, 2018). These secreted pro-inflammatory molecules recruit mast cells to aggregate, degranulate, etc. (Figure 8B). However, the following two issues still need to be verified by rigorous experiments: (1) Is there a true and continuous transmission of neural excitation from the plantar to the acupoint? (2) Whether the transmitted excitation results in the release of neurotransmitters necessary for local inflammatory responses? These are new research topics that require new research methods, especially *in vivo* studies.

## Data availability statement

The raw data supporting the conclusions of this article will be made available by the authors, without undue reservation.

## Ethics statement

The animal study was reviewed and approved by the Ethics Committee of the Laboratory Animal Center of Tongji University.

## Author contributions

X-FG and LX conceived and supervised the project. WL, JL, AC, DD, and TZ conducted the experiments. QL and JS guided the experimental implementation. X-FG wrote the manuscript with inputs from all authors.

## Supplementary material

Supplementary Figure 1 The CFA models were successfully built. An equal volume of saline was injected into the left hind paw plantar as the control. **(A)** The mechanical pain thresholds of the hind paw were reduced significantly by CFA treatment from day 1. *n* = 6, *t*-test, *P_*CFA vs. saline*_* < 0.0001 (D1). **(B)** The thermal pain thresholds of the hind paw were reduced significantly by CFA treatment from day 1. *n* = 6, *t*-test, *P_*CFA vs. saline*_* < 0.0001 (D1).

Supplementary Figure 2 CNO did not change the pain thresholds of plantar both in the CFA model and in normal mice without expression of M4 muscarinic DREADD receptors. **(A)** The mechanical pain thresholds and thermal pain thresholds of the ipsilateral hind paw plantar were not changed by CNO treatment in the CFA model. *n* = 6. paired *t*-test. For mechanical pain thresholds, *P* = 0.6109. For thermal pain thresholds, *P* = 0.0648. **(B)** The mechanical pain thresholds and thermal pain thresholds of the ipsilateral hind paw plantar were not changed by CNO treatment in normal mice. *n* = 6, paired *t*-test. For mechanical pain thresholds, *P* = 0.2374. For thermal pain thresholds, *P* = 0.4092.

Supplementary Figure 3 CNO did not change the pain thresholds of ST36 acupoint or LST both in normal mice and in the CFA model without expression of M3 muscarinic DREADD receptors. **(A)** The mechanical pain thresholds of the ipsilateral ST36 acupoint and the LST in the normal mice were not changed by CNO treatment. *n* = 6, paired *t*-test. For ST36 acupoint, *P* = 0.8053. For LST, *P* = 0.2892. **(B)** The mechanical pain thresholds of the ipsilateral ST36 acupoint and the LST in the CFA model were not changed by CNO treatment. *n* = 6, paired *t*-test. For ST36 acupoint, *P* = 0.3907. For LST, *P* = 0.4650.
