# Supplementary figures and images for: Shared nociceptive dorsal root ganglion neurons participating in acupoint sensitization
Source: Front Mol Neurosci. 2022 Aug 29;15:974007. doi: 10.3389/fnmol.2022.974007 (PMC9465389; doi:10.3389/fnmol.2022.974007)

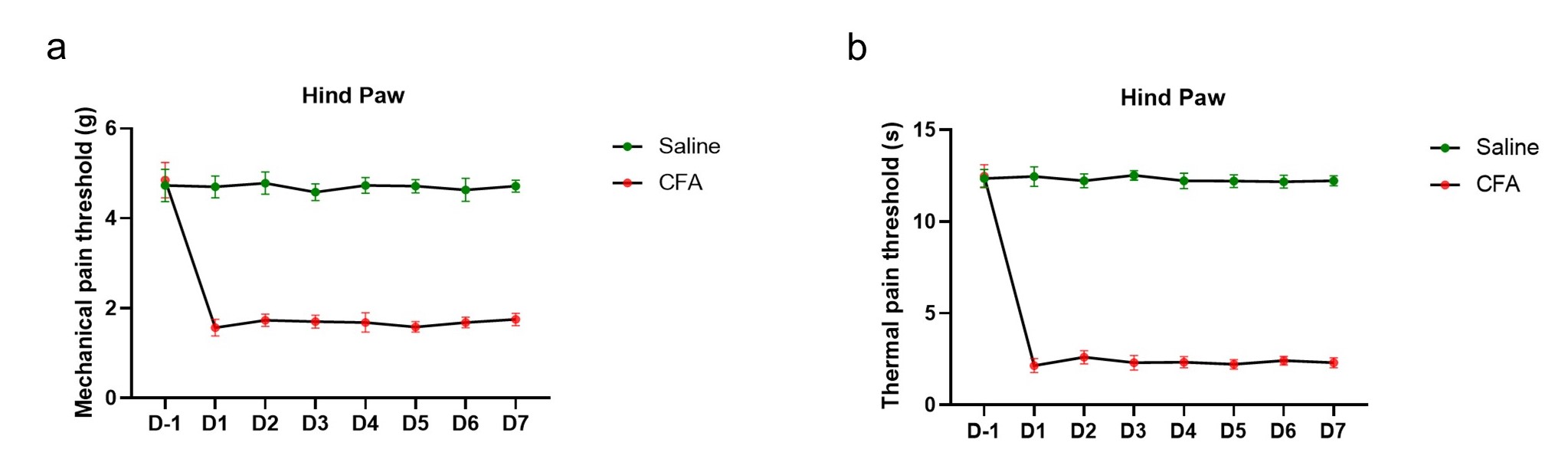

Supplement: Supplementary Figure 1 — The CFA models were successfully built. An equal volume of saline was injected into the left hind paw plantar as the control. (A) The mechanical pain thresholds of the hind paw were reduced significantly by CFA treatment from day 1. n = 6, t-test, PCFA vs. saline < 0.0001 (D1). (B) The thermal pain thresholds of the hind paw were reduced significantly by CFA treatment from day 1. n = 6, t-test, PCFA vs. saline < 0.0001 (D1). [file Image_1.jpeg]

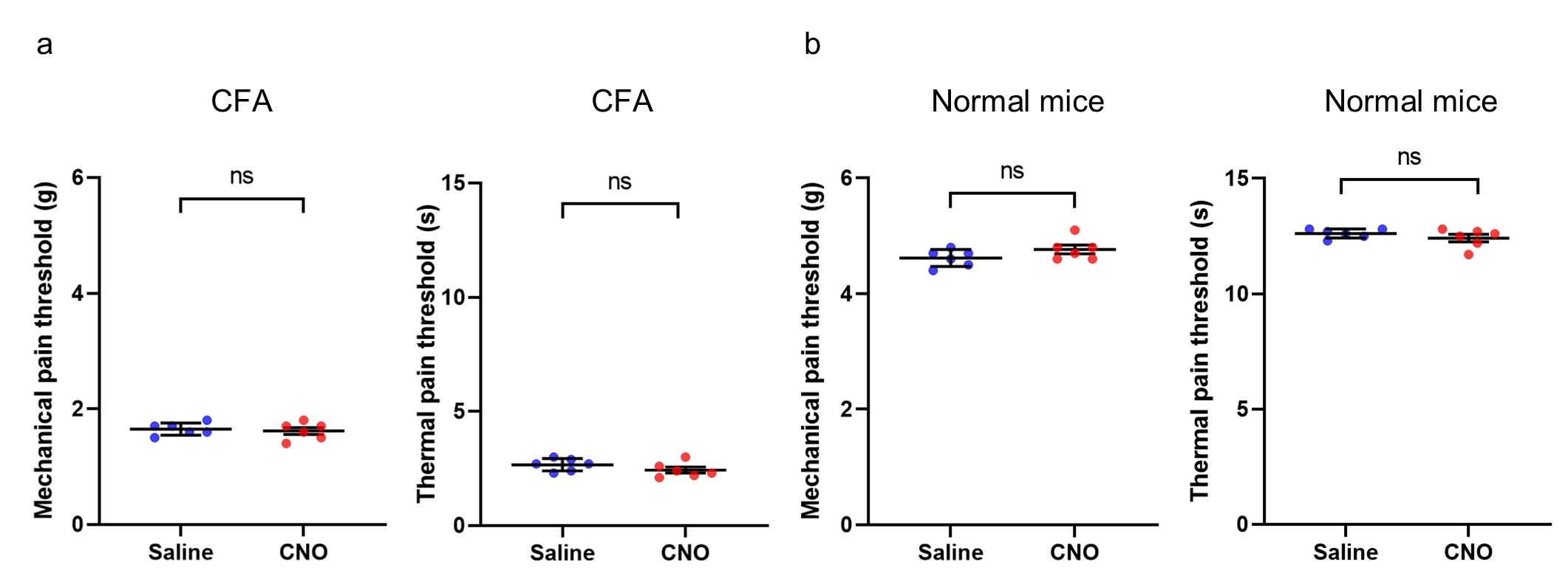

Supplement: Supplementary Figure 2 — CNO did not change the pain thresholds of plantar both in the CFA model and in normal mice without expression of M4 muscarinic DREADD receptors. (A) The mechanical pain thresholds and thermal pain thresholds of the ipsilateral hind paw plantar were not changed by CNO treatment in the CFA model. n = 6. paired t-test. For mechanical pain thresholds, P = 0.6109. For thermal pain thresholds, P = 0.0648. (B) The mechanical pain thresholds and thermal pain thresholds of the ipsilateral hind paw plantar were not changed by CNO treatment in normal mice. n = 6, paired t-test. For mechanical pain thresholds, P = 0.2374. For thermal pain thresholds, P = 0.4092. [file Image_2.jpeg]

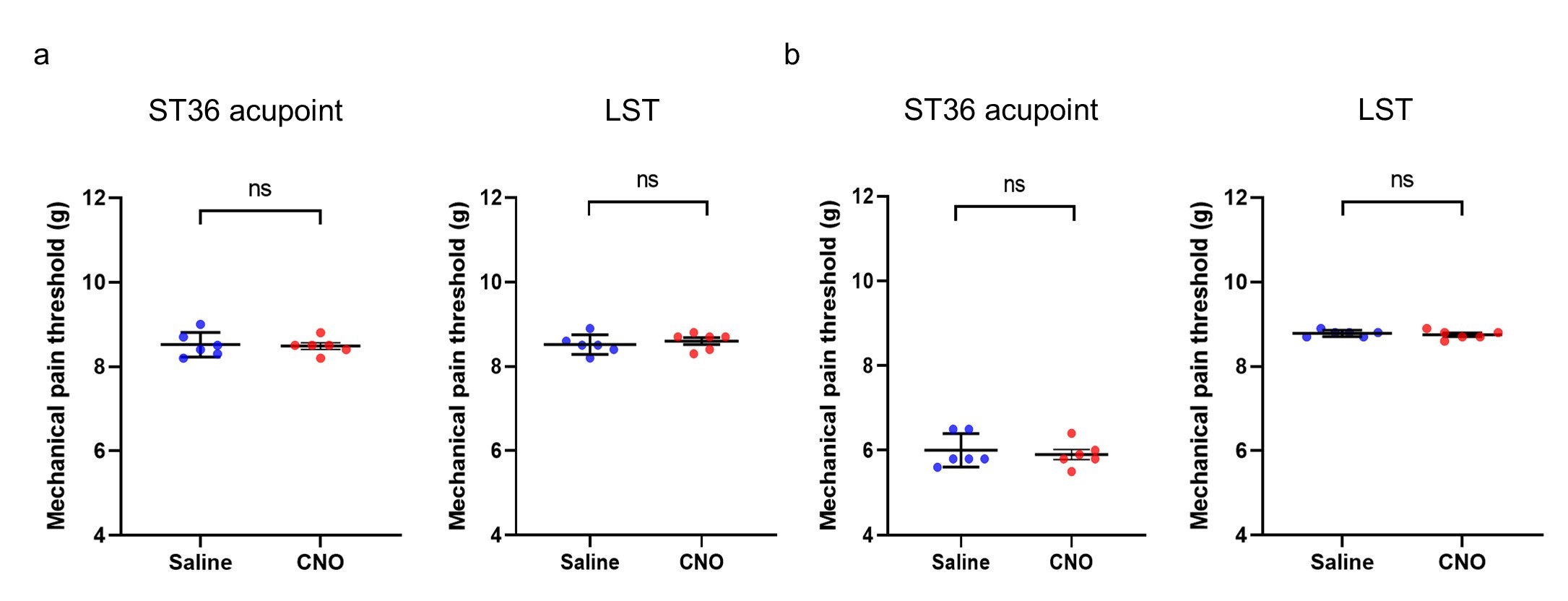

Supplement: Supplementary Figure 3 — CNO did not change the pain thresholds of ST36 acupoint or LST both in normal mice and in the CFA model without expression of M3 muscarinic DREADD receptors. (A) The mechanical pain thresholds of the ipsilateral ST36 acupoint and the LST in the normal mice were not changed by CNO treatment. n = 6, paired t-test. For ST36 acupoint, P = 0.8053. For LST, P = 0.2892. (B) The mechanical pain thresholds of the ipsilateral ST36 acupoint and the LST in the CFA model were not changed by CNO treatment. n = 6, paired t-test. For ST36 acupoint, P = 0.3907. For LST, P = 0.4650. [file Image_3.jpeg]
